# Forearm vasodilator responses to a *β*‐adrenergic receptor agonist in premenopausal and postmenopausal women

**DOI:** 10.14814/phy2.12032

**Published:** 2014-06-11

**Authors:** Ronee E. Harvey, Jill N. Barnes, Nisha Charkoudian, Timothy B. Curry, John H. Eisenach, Emma C. Hart, Michael J. Joyner

**Affiliations:** 1 Mayo Clinic College of Medicine, Rochester, Minnesota; 2 Department of Anesthesiology, Mayo Clinic, Rochester, Minnesota; 3 U.S. Army Research Institute of Environmental Medicine, Natick, Massachusetts; 4 School of Physiology and Pharmacology, University of Bristol, Bristol, UK

**Keywords:** *β*‐adrenoreceptor, endothelium, menopause

## Abstract

Beta‐adrenergic vasodilator responses may be blunted in humans who are at an increased risk for hypertension. Because menopause is associated with an increase in blood pressure, we tested the hypothesis that forearm blood flow responses to the *β*‐adrenergic receptor agonist isoproterenol are blunted in older, postmenopausal women compared to young, premenopausal women. We used venous occlusion plethysmography to measure forearm blood flow in young premenopausal (26 ± 1 years; *n* = 13) and postmenopausal (61 ± 2 years; *n* = 12) women. Forearm blood flow and mean arterial pressure were measured at baseline and during isoproterenol infusion at 1.0, 3.0, 6.0, and 12.0 ng/100 mL tissue/min. The two groups did not differ in body mass index or mean arterial pressure. Baseline forearm blood flow was similar between young and postmenopausal women (3.7 ± 0.5 vs. 2.9 ± 0.4 mL/100 mL tissue/min, respectively; *P* > 0.05). At the lowest dose of isoproterenol, forearm blood flow vasodilator responses were lower in postmenopausal women compared with young women (5.8 ± 0.4 vs. 7.4 ± 0.3 mL/100 mL tissue/min, respectively; *P* < 0.05). Thereafter, forearm blood flow remained similar between the groups for the remaining isoproterenol doses. In conclusion, *β*‐adrenergic receptor‐mediated forearm vasodilator responses are blunted in healthy, older postmenopausal women at lower but not higher doses of isoproterenol. This suggests that in aging women, *β*‐adrenergic receptor‐mediated vasodilator responses may be blunted at a moderate level of stimulation while maximum receptor responses are preserved.

## Abstract (precis)

This study assessed potential differences in *β*‐adrenergic receptor responsiveness to forearm isoproterenol infusion in young premenopausal and older postmenopausal women. Our data showed that *β*‐receptor vasodilator responses to low‐dose isoproterenol are blunted in postmenopausal women in comparison to young women, and responses to higher doses of isoproterenol are similar between the two groups. These results suggest that there is some attenuation of *β*‐receptor responsiveness in aging women while maximum vasodilator responses remain intact.

## Introduction

The risk of developing hypertension is greater in young and middle‐aged men than in women of the same age range (Burt et al. 1995). Although this risk increases with age in both sexes, women have a more dramatic rise in hypertension prevalence after the onset of menopause, and this may even surpass that of men (Cutler et al. 2008). Endothelial dysfunction, and specifically altered *β*‐adrenergic receptor responsiveness, has been found in men with borderline hypertension (Stein et al. 1995). In comparison to normotensive men, those with borderline hypertension have a lower absolute forearm blood flow response to local infusion of isoproterenol, a nonselective *β*‐adrenergic receptor agonist. Therefore, it is possible that the onset of menopause and related changes in blood pressure regulation are also associated with decreased *β*‐adrenergic receptor responsiveness.

We were interested in this topic for two reasons. First, Kneale et al. demonstrated that young premenopausal women had a lower percent change in forearm blood flow than young men during local infusion of norepinephrine (Kneale et al. 2000). Although norepinephrine has greater affinity for *α*‐adrenergic receptors, it also binds to *β*_2_‐adrenergic receptors. In this context, men and women demonstrated similar vasoconstriction to norepinephrine after local *β*‐adrenergic blockade. This suggested that sex‐specific differences in *β*_2_‐receptor affinity for norepinephrine that can offset *α*‐receptor‐mediated vasoconstriction. In this context, young women reportedly have higher *β*_2_‐adrenergic receptor sensitivity than young men, as women demonstrate a greater absolute change in blood flow in response to the infusion of the *β*_2_‐receptor agonist albuterol (Kneale et al. 2000). This contrast may be due to an interaction between the *β*‐receptors and female sex hormones in men and women.

Second, a recent study from our laboratory suggested that *β*‐adrenergic receptor sensitivity is different in young premenopausal women compared with older postmenopausal women (Hart et al. 2011). Similar to the study by Kneale et al., we found that in young women, there were greater changes in absolute and relative blood flow responses to local norepinephrine during systemic *β*‐receptor blockade with propranolol compared with before propranolol, again suggesting that *β*‐adrenergic receptor activity offsets the *α*‐receptor‐mediated vasoconstrictor response. However, in postmenopausal women, the vasoconstrictor response to norepinephrine was similar before and during *β*‐blockade (for both absolute and relative values), implying that the extent to which *β*‐adrenergic receptors oppose *α*‐adrenergic vasoconstriction was minimal in the postmenopausal group. Additionally, we reported that the balance between total peripheral resistance (TPR), muscle sympathetic nerve activity (MSNA), and mean arterial pressure (MAP) was altered by *β*‐blockade in young women but not older women. Taken together, this work suggests an important role of the *β*‐receptors in regulating vascular tone, and thus, blood pressure. However, function of the *β*‐receptors, or their affinity to respond to norepinephrine, is attenuated or lost in postmenopausal women, possibly contributing to the increased risk of hypertension in this group.

The studies by both Hart et al. and Kneale et al. suggested that *β*‐receptor sensitivity or responsiveness are different between young women and postmenopausal women; however, this hypothesis has not been directly tested. Therefore, we compared peripheral vasodilator responses to *β*‐adrenergic receptor stimulation between young premenopausal and older postmenopausal women using the nonselective *β*‐adrenergic receptor agonist isoproterenol. We tested the hypothesis that postmenopausal women have a blunted forearm vasodilator response to increasing doses of isoproterenol in comparison to young women.

## Materials and Methods

### Ethical approval

This study was approved by the Mayo Clinic Institutional Review Board. All subjects provided written informed consent prior to study participation.

### Subjects

Thirteen young premenopausal (age 26 ± 1 years) and 12 older postmenopausal (61 ± 2 years) women completed the study. Subjects were normotensive, nondiabetic, nonobese (body mass index <30 kg/m^2^), nonsmokers, and free of cardiovascular and chronic diseases. Subjects were not taking medications with the exception of oral contraceptives in young women (*n* = 12) and medication used to treat hypothyroidism (*n* = 1) and osteoporosis (*n* = 1) in postmenopausal women. All young women were studied during the early follicular phase of the menstrual cycle or during the low‐hormone phase of oral contraceptive use. Pregnant women were excluded from the study, and this was confirmed by a urine pregnancy test within 48 h prior to the study day. Postmenopausal women taking hormone replacement therapy were excluded. Postmenopausal was defined as at least 12 months since last menstrual period (Gracia et al. 2005).

### Experimental protocol

Subjects refrained from alcohol, caffeine, and exercise for 24 h prior to the study and following an overnight fast, participants were admitted to the Clinical Research Unit at the Mayo Clinic. Subjects rested in the supine position during instrumentation and throughout the study. A 20‐gauge, 5‐cm catheter was inserted into the brachial artery of the nondominant arm using aseptic technique. The arm was positioned at the level of the heart for the duration of the study. A pressure transducer level (model PX600F, Edwards Lifescience, Irvine, CA) was connected to the catheter to measure continuous beat‐to‐beat blood pressure. Heart rate was recorded continuously using a 3‐lead ECG (Cardiocap/5; Datex‐Ohmeda, Louisville, CO).

Forearm blood flow (FBF) was measured using mercury‐in‐silastic strain‐gauge venous occlusion plethysmography (Hokanson, Bellevue, WA) (Greenfield et al. 1963; Casey et al. 2008). Forearm volume was measured using water displacement. A blood pressure cuff was placed around the wrist of the nondominant hand and inflated to suprasystolic pressure to exclude circulation of the hand in the measurement. Another cuff was placed around the upper arm and cyclically inflated to 50 mmHg then deflated to obtain one blood flow every 15 s. FBF was measured for 2 min at baseline during saline infusion and for 2 min during an intra‐arterial isoproterenol hydrochloride (Isuprel^®^, Hospira, Lake Forest, IL) infusion at each of the following doses: 1.0, 3.0, 6.0, and 12.0 ng/100 mL tissue/min (Eisenach et al. 2002; Garovic et al. 2003). The infusions were adjusted for forearm volume and delivered via the brachial artery catheter using a calibrated, multisyringe infusion pump (model 55‐2219, Harvard Apparatus, South Natick, MA).

### Data analysis

Data were collected at 250 Hz and stored via an offline computer using the WinDaq (DATAQ Instruments, Akron, OH) software system. FBF was determined from the first derivative of the plethysmography recording during venous occlusion. FBF data are shown as averages of the highest three peak flows during each dose of isoproterenol infusion. MAP was derived from the arterial pressure waveform during FBF recording. Responses were analyzed as absolute values and percent changes in FBF and FVC in comparison to baseline (saline infusion) recordings. FBF was expressed as mL/100 mL tissue/min. Forearm vascular conductance (FVC) was calculated as FBF/MAP × 100 and expressed as mL/100 mL tissue/min/mmHg. Area under the curve (AUC) for FBF and FVC was assessed by calculating total FBF and FVC responses (at baseline and all isoproterenol doses) for each individual and determining the average AUC for each study group (young women and postmenopausal women). FBF and FVC AUC's are expressed as arbitrary units (AU).

### Statistical analysis

All group data are reported as mean ± SEM. Demographic data and baseline hemodynamic variables of the two groups were compared using the Student's *t*‐test (SigmaPlot 12.0, Systat Software, San Jose, CA). Inter‐ and intragroup forearm blood flow data, including FBF, FVC, MAP, percent changes in these values, and AUC for FBF and FVC, were also evaluated using the Student's *t*‐test. Two‐way repeated‐measures analysis of variance and Tukey's post hoc test for multiple comparisons were completed to assess for potential group × time (i.e., menopausal status × isoproterenol dose) interaction. A calculated *P*‐value of <0.05 was considered significant.

## Results

Demographic and baseline hemodynamic data for both young and postmenopausal (PM) women are summarized in Table 1. Forearm volume was greater in young women than PM women (823 ± 22 vs. 740 ± 21 mL, respectively; *P* < 0.05). PM women had greater baseline systolic blood pressure (young vs. PM, 127 ± 4 vs. 141 ± 4 mmHg; *P* < 0.05), pulse pressure (young vs. PM, 53 ± 2 vs. 70 ± 4 mmHg; *P* < 0.01), and norepinephrine levels (young vs. PM, 123 ± 14 vs. 191 ± 17 pg/mL; *P* < 0.01). Body mass index, baseline heart rate, and resting MAP were similar between the two groups.

**Table 1.** Demographic and baseline characteristics of study subjects

Demographics	Young (*n* = 13)	PM (*n* = 12)
Age, years	26 ± 1 (22–29)	61 ± 2* (54–76)
Height, cm	167.0 ± 1.7 (158–178)	164.1 ± 1.8 (153–178)
Weight, kg	67.4 ± 2.2 (54–77)	63.6 ± 1.7 (55–72)
BMI, kg/m^2^	24.2 ± 0.7 (20–30)	23.7 ± 0.7 (20–27)
Forearm volume, mL	823 ± 22 (700–980)	740 ± 21* (650–840)
Heart rate, bpm	63 ± 3 (44–83)	58 ± 3 (42–76)
Systolic blood pressure, mmHg	127 ± 4 (108–152)	141 ± 4* (119–165)
Diastolic blood pressure, mmHg	74 ± 2 (62–82)	71 ± 2 (63–85)
Pulse pressure, mmHg	53 ± 2 (40–71)	70 ± 4** (51–95)
Mean arterial pressure, mmHg	92 ± 2 (80–104)	95 ± 2 (82–109)
Norepinephrine, pg/mL	123 ± 14 (53–228)	191 ± 17** (102–283)
Epinephrine, pg/mL	43 ± 10 (10–124)	43 ± 6 (15–82)
Estradiol, pg/mL	16.1 ± 5.5* (2.5–66)	3.9 ± 0.5* (2.5–8.4)
Progesterone, ng/mL	0.52 ± 0.07 (0.15–0.93)	0.19 ± 0.02** (0–0.31)
Testosterone, ng/dL	21.7 ± 1.8 (11.0–34.0)	13.9 ± 1.7** (7.0–23.0)

Mean ± SEM (minimum–maximum); PM, postmenopausal.

* *n* = 12.

*P < 0.05, **P < 0.01.

**Figure 1.**
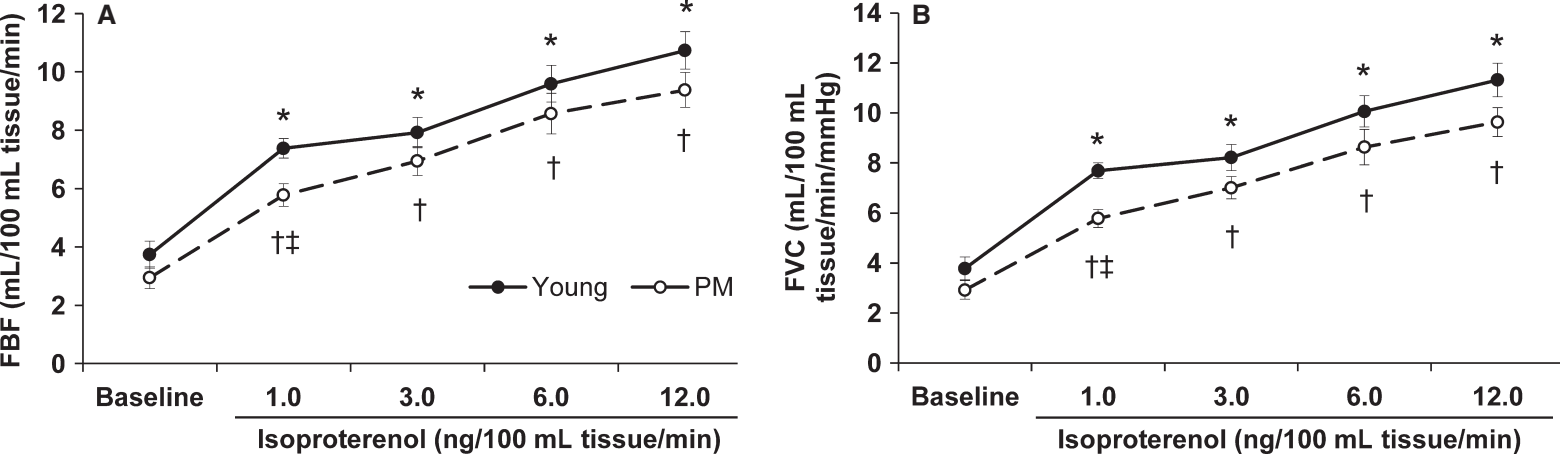
Forearm blood flow (FBF, [A]) and forearm vascular conductance (FVC, [B]) values at baseline and with increasing doses of isoproterenol in young women and postmenopausal (PM) women. Mean ± SEM. *Data significantly different from baseline in young women (*P* < 0.05). ^†^Data significantly different from baseline in PM women (*P* < 0.05). &ddagger;Data significantly different from young women (*P* < 0.05).

Forearm blood flow, MAP, and FVC results for the two groups of women at baseline and for all doses of isoproterenol are shown graphically in Figure 1. Within each group, FBF and FVC values increased significantly from baseline in both young and PM women with all doses of isoproterenol (*P* < 0.05). At baseline, FBF was 3.7 ± 0.5 mL/100 mL tissue/min for young women and increased to 10.7 ± 0.6 mL/100 mL tissue/min at the 12.0 ng/100 mL tissue/min dose of isoproterenol. Baseline FVC was 3.8 ± 0.5 mL/100 mL tissue/min/mmHg and rose to 11.3 ± 0.7 mL/100 mL tissue/min/mmHg in young women. In PM women, FBF at baseline was 2.9 ± 0.4 mL/100 mL tissue/min and increased to 9.4 ± 0.6 mL/100 mL tissue/min at the highest dose of isoproterenol, while baseline FVC equaled 2.9 ± 0.4 mL/100 mL tissue/min/mmHg and rose to 9.6 ± 0.6 mL/100 mL tissue/min/mmHg. Percent changes in FBF and FVC values increased significantly within each group for all doses of isoproterenol (*P* < 0.05).

At baseline, FBF, MAP, and FVC did not differ between young and PM women. At the first isoproterenol dose (1.0 ng/100 mL tissue/min), FBF and FVC were significantly greater in young women, where FBF was 7.4 ± 0.3 mL/100 mL tissue/min for young women and 5.8 ± 0.4 mL/100 mL tissue/min for PM women (*P* < 0.05), and FVC was 7.7 ± 0.3 vs. 5.8 ± 0.4 mL/100 mL tissue/min/mmHg (young vs. PM women, respectively; *P* < 0.001). Percent changes in FBF (*P* = 0.791) and FVC (*P* = 0.686) were not significantly different between the groups. All FBF and FVC values remained similar between the two groups for the remaining doses of isoproterenol. There was no significant menopausal status × isoproterenol dose interaction for FBF (*P* = 0.75), FVC (*P* = 0.62), percent change in FBF (*P* = 0.50), or percent change in FVC (*P* = 0.57).

Forearm blood flow AUC was similar between young and PM women (108.1 ± 6.0 vs. 94.2 ± 6.5 AU, respectively; *P* = 0.13). AUC for FVC tended to be greater in young women than in PM women (113.2 ± 5.9 vs. 95.4 ± 6.3 AU, respectively; *P* = 0.051).

## Discussion

The primary findings of this study are (1) with increasing doses of isoproterenol infusion, both young premenopausal women and older postmenopausal women have a significant increase in vasodilation measured by forearm blood flow; (2) at a low dose of isoproterenol, forearm blood flow responses are blunted in postmenopausal women in comparison to young women; and (3) at higher doses of isoproterenol, vasodilator responses are similar between the two groups of women. Previous studies suggest sex differences in FBF responses to *β*_2_‐adrenergic receptor stimulation in young adults (Kneale et al. 2000). Our present results suggest that sex hormone levels, which are dramatically reduced at menopause, can contribute to lower vasodilator capacity in aging women. Beta‐receptor responsiveness at a lower dose of isoproterenol was attenuated, whereas maximum vasodilator capacity to *β*‐adrenergic receptor stimulation was preserved in postmenopausal women despite dramatic differences in sex hormone levels.

The results from our current study are somewhat surprising in light of previous findings from our laboratory by Hart et al. (2011), which showed that *β*‐receptor blockade augments the effects of *α*‐adrenergic vasoconstriction at different doses of norepinephrine infusion in young women. This may be because blocking the *β*‐receptors prevents any vasodilation that could offset *α*‐receptor‐mediated vasoconstriction in this population. In postmenopausal women, absolute and relative changes in blood flow in response to norepinephrine were similar before and during *β*‐receptor blockade, suggesting decreased sensitivity to the vasodilator influence of *β*‐receptors. It would be expected that the differences in *β*‐receptor sensitivity seen in these previous studies would also hold true for receptor agonist activity at all levels of isoproterenol. We report that *β*‐adrenergic vasodilator responsiveness is decreased in postmenopausal women with infusion of low‐dose isoproterenol, but it remains similar to that of young women with higher doses of the drug. It is possible that results of these studies differ for several reasons. First, in the Hart et al. (2011) *β*‐blockade study, propranolol drug administration was delivered systemically and norepinephrine was infused locally into the forearm, while in the current study, isoproterenol was infused locally into the forearm. Also, we did not perform isoproterenol infusion under both baseline and *β*‐blockade conditions to reproduce the exact conditions of the Hart et al. study. In addition, MSNA was not measured during isoproterenol infusion in this study. Baseline measurements demonstrate that serum levels of norepinephrine are significantly higher in postmenopausal women in comparison to young women (191 ± 17 vs. 123 ± 14, respectively; *P* < 0.01), and this may be reflective of greater sympathetic tone and/or altered norepinephrine clearance. However, we cannot confirm this in the present study and no conclusions can be made concerning the degree by which MSNA may be acting on the vessel externally to counteract the internal stimulation of *β*‐receptors.

In addition, the study by Hart et al. (2009, 2011) demonstrated that *β*‐receptors play an important role in regulating the relationships among MSNA, TPR, and MAP. At baseline, MSNA and TPR were not correlated in young women. However, during *β*‐blockade, MSNA was positively associated with TPR and MAP, meaning that *β*‐adrenergic receptors may prevent the transduction of neural signals from directly affecting TPR in young women, thereby preventing MSNA from directly increasing MAP. In postmenopausal women, MSNA, TPR, and MAP were positively related before and after *β*‐blockade, showing that *β*‐adrenergic receptor activity has no (or only minimal) effect on these parameters, and therefore may be less important in blood pressure control in this group. Overall, previous data support the concept that *β*‐adrenergic receptor activity is important in regulating both peripheral vasoconstriction and peripheral blood flow, thereby affecting resting arterial pressure in young women, and that this regulatory mechanism is lost or attenuated with aging and the onset of menopause.

This study takes into account how *β*‐receptor function in the vasculature is altered with both aging and menopause. While in a different aspect, Sherwood et al. studied cardiac *β*‐adrenergic receptor sensitivity in postmenopausal women in comparison to age‐matched premenopausal women (Sherwood et al. 2010). The study estimated *β*‐receptor sensitivity based on the chronotropic dose of intravenous isoproterenol needed to raise heart rate by 25 bpm and found that cardiac *β*‐adrenergic receptor sensitivity was decreased in postmenopausal women. In contrast to our forearm model, both *β*_1_‐ and *β*_2_‐receptors were being assessed and the decrease in receptor sensitivity may have been mostly due to cardiac *β*_1_‐adrenergic changes, as it has been shown that *β*_1_‐receptor effects on heart rate decrease with aging (Fleg 1986). Taken together with our own data, it appears that *β*‐receptor sensitivity or responsiveness may decrease not only in the blood vessels but also in multiple portions of the cardiovascular system with aging and menopause.

A limitation to the interpretation of our present results is that the provided doses of isoproterenol may or may not reflect the level of endogenous *β*‐adrenergic receptor ligand (i.e., norepinephrine) circulating throughout the body under various physiological states. It is possible that the lowest dose of isoproterenol (1.0 ng/100 mL tissue/min) is reflective of physiological vasodilation, and that the higher doses are supraphysiological. This would suggest that the deficit seen in postmenopausal women in this study is physiologically relevant. However, it is difficult to confirm which doses of isoproterenol mimic normal physiological responses. The divergence in FBF and FVC between young and postmenopausal women was noted only at the 1.0 ng/100 mL tissue/min dose of isoproterenol, and it is possible that this drug dose is more reflective of the early stimulation of *β*‐receptors in vivo prior to complete receptor saturation. We adjusted isoproterenol infusion concentration for each individual based on forearm volume, which was measured using water displacement. Potentially, body fat composition differed between young and postmenopausal women, as forearm volume was greater in the group of young women. Alternatively, imaging could have been used to determine fat and fat‐free mass of the forearm for isoproterenol dose calculation. However, body mass index was similar between the two groups of women, so it is unlikely that slight variations in forearm fat mass may have influenced our results. Additionally, our present study was not designed to evaluate cellular or molecular mechanisms at the level of the vascular smooth muscle or endothelium. In order to obtain further information on such mechanisms, future studies could specifically evaluate receptor number or density of relevant tissue samples from young and postmenopausal women. Additionally, *β*‐adrenergic receptor responsiveness to isoproterenol can be affected by genetic polymorphisms (Garovic et al. 2003; Eisenach et al. 2006), sodium intake (Eisenach et al. 2006), and ability to respond to adenosine infusion (Martin et al. 2006), which we did not control for experimentally in the present study.

In summary, we have demonstrated that the *β*‐adrenergic receptor‐mediated vasodilator response to low‐dose isoproterenol is blunted in healthy postmenopausal women in comparison to young premenopausal women. This suggests that *β*‐receptor responsiveness may be moderately diminished, while maximum vasodilator capacity is maintained, with aging in healthy, normotensive postmenopausal women. Better understanding of the effect of age on *β*‐adrenergic receptors remains a clinically relevant issue in order to establish which changes in vascular function could act as biomarkers of cardiovascular risk. This is particularly important as altered adrenergic receptor function (with advancing age or after menopause) may contribute to the risk of developing cardiovascular disease, in particular hypertension. In addition, increased understanding of the physiological role of the *β*‐receptor in the cardiovascular system and the elevation of blood pressure in postmenopausal women may reveal an opportunity for alternative therapeutic targets in hypertension.

## Acknowledgments

We thank Shelly Roberts, Sarah Wolhart, Christopher Johnson, Casey Hines, Luke Matzek, Jennifer Taylor, and Pam Engrav for their assistance in the conduction of the studies. We also thank the volunteers who participated in the study. The views, opinions, and/or findings contained in this article are those of the authors and should not be construed as an official Department of the Army position, or decision, unless so designated by other official documentation. Approved for public release; distribution unlimited.

## Conflict of Interest

None declared.
